# A Self-Powered Wearable Motion Sensor for Monitoring Volleyball Skill and Building Big Sports Data

**DOI:** 10.3390/bios12020060

**Published:** 2022-01-24

**Authors:** Weijie Liu, Zhihe Long, Guangyou Yang, Lili Xing

**Affiliations:** 1 School of Physics, University of Electronic Science and Technology of China, Chengdu 611731, China; liuwjie@std.uestc.edu.cn (W.L.); yanggy127@163.com (G.Y.); 2 Department of Mechanical Engineering, City University of Hong Kong, 83 Tat Chee Avenue, Kowloon, Hong Kong SAR 999077, China; zh.long@my.cityu.edu.hk

**Keywords:** self-powered, motion monitoring, wearable electronics, big sports data

## Abstract

A novel self-powered wearable motion sensor for monitoring the spiking gesture of volleyball athletes has been manufactured from piezoelectric PVDF film. The PVDF film can convert body mechanical energy into electricity through the piezoelectric effect, and the flexible device can be conformably attached on the hand or arm. The sensor can work independently without power supply and actively output piezoelectric signals as the sports information. The sensor can detect the tiny and fine motion of spiking movement in playing volleyball, reflecting the skill. Additionally, the sensor can also real-time monitor the pulse changes and language during a volleyball match. The self-powered sensors can link to a wireless transmitter for uploading the sports information and building big sports data. This work can provoke a new direction for real-time sports monitoring and promote the development of big sports data.

## 1. Introduction

In recent decades, volleyball has been very popular due to its competitive and technical features and has attracted much attention at the Olympic Games. As the most active and effective offensive method in volleyball matches, spiking plays the most critical role in the completion of offensive tactics [1,2,3]. The movement of the athletes’ fingers and arms determines the quality and accuracy of the spike. Therefore, monitoring the movements is of great significance for improving skill in volleyball. At present, the commonly used method for athletes’ sports training and monitoring is videography, which has certain limitations [4,5]. For example, high-speed cameras manifest susceptibility to environmental factors, such as light and the movement of multiple athletes, which can easily distort the results captured by the camera [6]. Another common limitation of video-based systems is the heavy computer load. The images acquired by high-speed cameras require a large amount of storage, and the image analysis demands a huge amount of calculation. This greatly increases the expense of purchasing equipment and the cost of its maintenance. In addition, the camera cannot provide real-time monitoring of the athlete’s exercise intensity and changes in the bending angle of the fingers and elbows.

With the development of wearable electronics, an alternative solution is using portal inertial sensors [7,8,9]. By establishing an inertial information database for athletes who use sensors, motion characteristics can be extracted from sensing data. However, the power supply of these wearable devices has become one of the bottlenecks in their further development. Rechargeable batteries need to be charged frequently, which leads to great inconvenience to the real-time collection of athletes’ daily training data. The emerging self-powered sensors provide new ideas for overcoming this problem [10,11,12,13,14,15]. Recently, self-powered flexible biosensing devices have been used in real-time monitoring various physiological indicators like blood glucose, body temperature, heart rate and pulse in daily life [16,17,18,19,20,21,22,23,24,25]. These self-powered flexible sensors that possess the ability to work without external power could also be probably used in the domain of sports motion monitoring [26,27,28,29,30,31,32,33].

In this work, a self-powered wearable sensor has been fabricated from piezoelectric PVDF film. The working mechanism is based on the piezoelectric effect upon applied deformation. The device can monitor the finger and arm movements during spiking without an external power supply by collecting the mechanical energy of body activity. The sensor has excellent performance in terms of flexibility and collects sensing information without affecting the normal activities of the athletes’ limb. Besides, the mechanical energy of the human body can be converted into electrical energy to charge capacitors and provide power for wireless transmitters such as Bluetooth devices. In practical applications, the sensor can detect the athlete’s spiking state and wirelessly transmit physiological information for building big sports data. With the exception of monitoring the spiking posture, the sensor can also monitor other sports indicators, such as the pulse. This research has great potential in realizing efficient volleyball training and big sports data.

## 2. Materials and Methods

### 2.1. Device Fabrication

Two grams of PVDF powder (purchased from Chengdu Keweizhuo Technology Co., Ltd., Chengdu, China) was dissolved in a mixed solution of 12 mL dimethylformamide and 18 mL acetone. The mixture was sealed and then stirred with a magnetic stirrer for 12 h to form a uniform slurry. The PVDF film was obtained after the PVDF slurry was dried at 60 °C for 12 h at least. Next, the PVDF film was polarized under a 20 kV/mm electric field in a silicone oil bath at 80 °C. The silicone oil was removed with ether. Copper electrodes were then pasted on both surfaces of the PVDF film. A PDMS mixture of AN elastomer base and curing agent (with a mass ratio of 10:1) after ultrasound treatment for 15 min to eliminate air bubbles was used to package the PVDF film as a protective layer. The PDMS encapsulation layer could prevent the sensor from being influenced by the sweat on the skin, improving the durability of the sensor. The film encapsulated by PDMS was of good flexibility and could be completely attached to the skin without affecting the movement of the limb.

### 2.2. Characterization and Measurement

The morphology and structure of the device were investigated by scanning electron microscope (SEM, Zeiss Gemini 300). Two copper foils were glued to the electrodes of the PVDF film for piezoelectric measurement. External force was applied to the edge of the sensor through a programmable stepper motor, causing the device to bend and deform. The bending angle of the sensor was controlled by adjusting the operating position of the stepping motor. Similarly, the operating frequency of the stepper motor could be changed by programming during the measurement. The outputting piezoelectric voltage of the sensor was recorded by a low-noise preamplifier (Model SR560, Stanford Research Systems).

## 3. Results and Discussion

**Figure 1:**
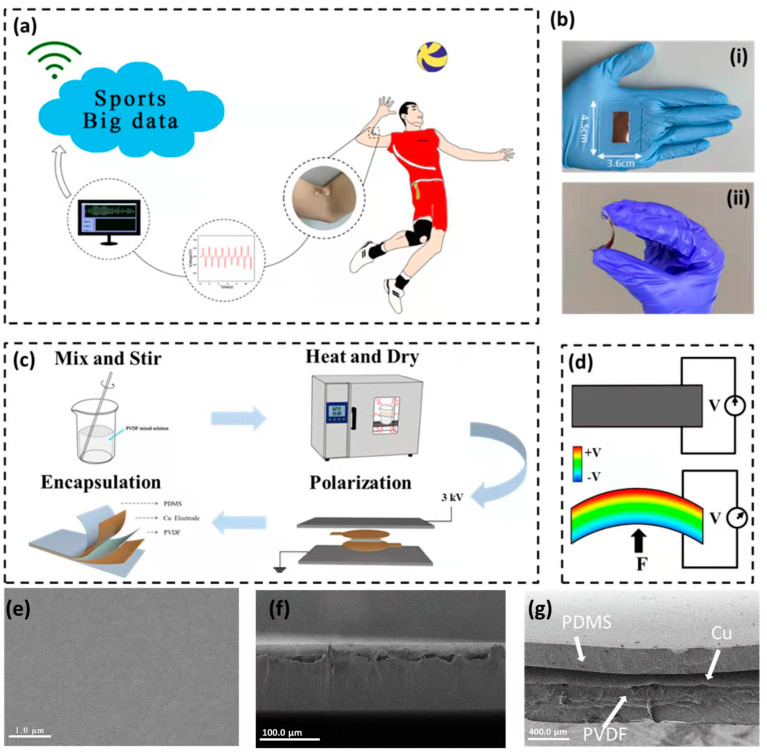
Experimental design of self-powered wearable motion sensor for monitoring volleyball skill and building big sports data. (**a**) The wearable sensor for building big sports data; (**b**) the optical image of sensor; (**c**) The fabrication processes of the self-powered sensor; (**d**) The working mechanism of the piezoelectric sensor. (**e**) The top-view SEM image of PVDF film; (**f**) The side-view SEM image of PVDF film; (**g**) The side-view SEM image of PVDF film encapsulated by PDMS.

The experimental design, optic imaging, material characterization, fabrication procedure and working mechanism of the self-powered wearable motion sensor for monitoring volleyball skill and building big sports data are shown in Figure 1. Figure 1a shows the experimental design of the self-powered wearable sensors for building big sports data. The self-powered wearable sensors can be conformably attached on athletes’ finger and elbow and can, in real time and continuously, monitor exercise parameters during volleyball training or match. The sensing signals can be uploaded for building big sports data through the wireless transmitter. The optical photographs of the sensor are shown in Figure 1b. The size of the sensor is 4.5 × 3.6 cm (Figure 1b(i)). Figure 1b shows that the sensor has good flexibility and can be easily bent by human motion. In order to prevent the PVDF film from being damaged, PDMS was used to encapsulate the whole device. Figure 1c schematically illustrates the fabrication process. The detailed processes of preparing PVDF film, polarizing the film and encapsulating with PDMS can be found in the experimental section.

The working mechanism is shown in Figure 1d [34,35,36]. In the static state, the arrangement of dipoles in PVDF is orderly. Due to the influence of the built-in electric field, there are a lot of bound charges on the surface. As external force is applied to the sensor (causing deformation), the direction of the dipole changes and the built-in electric field decreases. The surface charge will be released accordingly, and the released charge can be detected through the external circuit. Similarly, when the external force disappears, the dipole returns to its original state, and the released charges return to the surface of the PVDF. At the same time, the opposite signal against the previous one can be detected. The top-view and side-view SEM images of the sensor are shown in Figure 1e and Figure 1f, respectively. The thickness of PVDF film is ~109 μm. As shown in Figure 1g, PVDF film with copper electrodes on both sides is encapsulated by PDMS.

**Figure 2:**
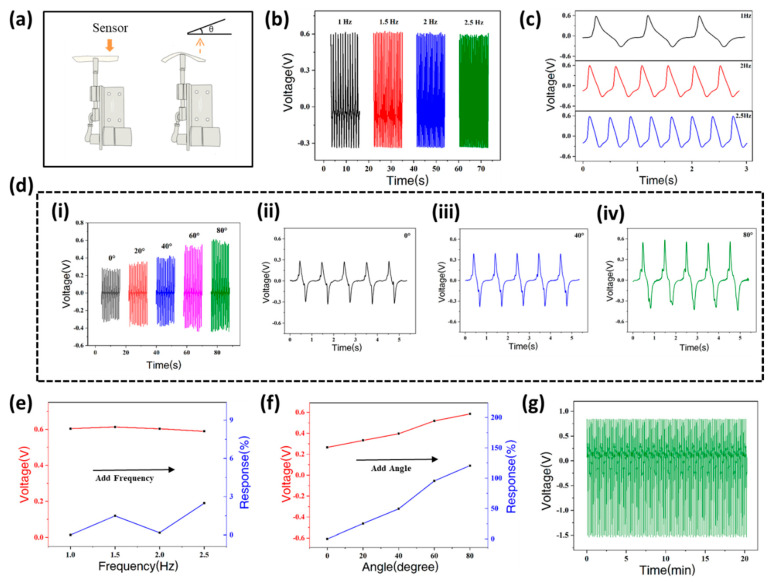
The performance of the self-powered wearable sensor. (**a**) The schematic diagram of the bending sensor with stepper motor; (**b**) The output piezoelectric voltage at different bending frequencies; (**c**) Details of output piezoelectric voltage at different frequencies; (**d**) The output piezoelectric voltage at different bending angles; (**e**) The output piezoelectric voltage response at different frequencies; (**f**) The output piezoelectric voltage response at different angles; (**g**) The durability test of sensor.

The performance of the self-powered flexible sensor under different experimental conditions is shown in the Figure 2. A programmable stepping motor applies force on the middle of the sensor, providing bending deformation. This process mimics the movement of knuckles and elbow joints upon spiking (Figure 2a). The frequency and angle of bending deformation can be controlled by adjusting the stepper motor. Figure 2b shows the piezoelectric output (voltage) of the sensor at different bending frequencies (under the same bending angle). When the frequency is 1, 1.5, 2 and 2.5 Hz, the output voltage is 0.604, 0.613, 0.603 and 0.589 V, respectively. It can be seen that the piezoelectric output is basically unchanged at different bending frequencies. The frequency of the output voltage signal is determined by the bending frequency. Figure 2c presents the enlarged details of the signal under different bending frequencies (1, 2, 2.5 Hz). Figure 2d(i) shows the output piezoelectric voltage with increasing bending angle (under the same frequency). When the bending angle is 0°, 20°, 40°, 60° or 80°, the piezo-voltage is 0.265, 0.332, 0.396, 0.518 and 0.585 V, respectively. Obviously, as the bending angle increases, the piezoelectric output of the sensor increases. Figure 2d(ii–iv) depict the details of the acquired signals at the bending angles of 0°, 40°, 80°.

The output piezoelectric voltage response of the sensor at different bending frequencies is shown in Figure 2e. The response of the sensor can be calculated with the following equation:

(1) $$R\%=\left|\frac{V_{0}-V_{i}}{V_{0}}\right|\times 100\%,\;$$

where $V_{0}$ is the output voltage at 1 Hz or 0°, and $V_{i}$ is the output voltage at other frequencies or angles. When the frequency is 1, 1.5, 2 or 2.5 Hz, the response is 0.00%, 1.49%, 0.17% and 2.48%, respectively. Clearly, the piezoelectric output of the sensor is quite stable and remains unchanged with the frequency. As shown in Figure 2f, the response of the piezoelectric output is positively correlated with the bending angle. When the bending angle is 0°, 20°, 40°, 60° or 80°, the response is 0.00%, 25.28%, 49.43%, 95.47% and 120.75%, respectively. It can be concluded that the sensor is of great suitability for monitoring the changing angles of human joints during bending. Volleyball is a sport that requires continuous competition and training for long periods of time and so suggests new requirements on the durability of sensors. The sensor presents superior durability in tests that last for 20 min (Figure 2g).

**Figure 3:**
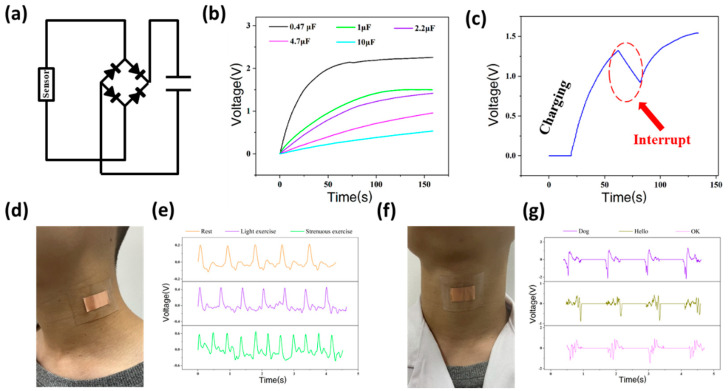
Multiple functions of the sensor. (**a**) Circuit diagram of the charging system for the sensor; (**b**) The charging capability of the sensor under different capacitance capacities; (**c**) The relationship between charging the voltage and charging time of sensor; (**d**) An optical image of the sensor while monitoring pulse; (**e**) The output piezoelectric voltage under volunteer’s different states; (**f**) The sensor for voice recognition; (**g**) The output piezoelectric voltage when the volunteer speaks different words.

Figure 3 shows the various functions of the piezoelectric effect-based sensor. The self-powered sensor has high electrical output performance. Under continuous press, the sensor can charge a capacitor to power Bluetooth or other wireless electronic devices (Figure 3a). Figure 3b shows that the capacitance (0.47–10 µF) has a significant impact on the charging performance of the sensor. The 0.47-µF capacitor can be charged to 2.13 V in 70 s, and the 1-µF capacitor can be charged to merely 1.18 V in the same time. When the charging process is interrupted, the voltage of the capacitor slowly decreases. The capacitor can still be charged up again, recovered by continuously tapping on the sensor (Figure 3c).

In addition to monitoring the spike posture, the sensor can also be designed to monitor other physiological indicators such as the pulse (Figure 3d). Figure 3e shows the pulse data of volunteers tested with the sensor in three different states: rest, light exercise, and strenuous exercise. In these three states, the volunteer’s pulses are about 70, 91, and 146 respectively, indicating the application of the sensor in the area of health monitoring. Through the real-time monitoring of the pulse, the sensor can realize the evaluation of exercise intensity, providing digitized exercise results, effectively avoiding accidents caused by exercise intensity exceeding the load of the heart and body. The sensor also has potential application in voice recognition. As shown in the Figure 3f,g, the sensor is attached to the volunteer’s throat to collect vocal signals. As volunteer’s utter different words (dog, hello, and OK), the output signal presents a unique waveform, revealing that the sensor has a certain degree of voice recognition function.

**Figure 4:**
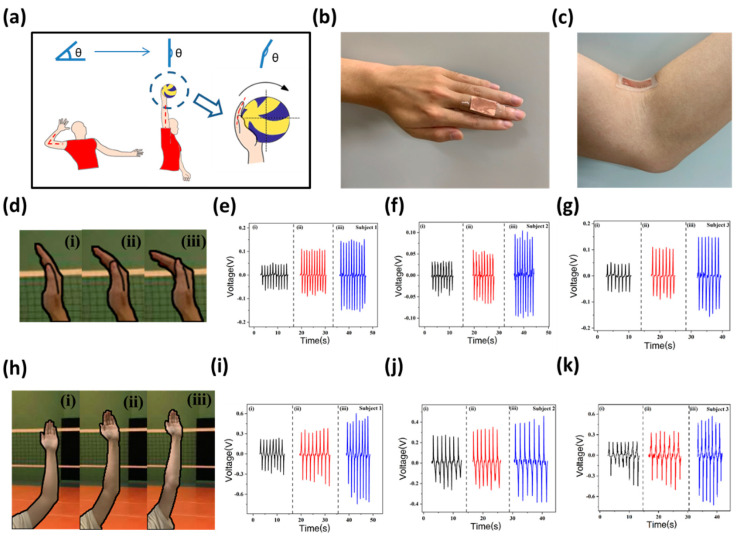
Practical application of the sensor. (**a**) A schematic diagram of spiking technology; (**b**)The optical image of the sensor attached on the finger; (**c**) An optical image of the sensor attached on the elbow; (**d**) A schematic diagram of different bending angles of palm during test; (**e**–**g**) The output piezoelectric voltage of three subjects when finger bending angle changes; (**h**) A schematic diagram of different bending angles of the elbow during testing; (**i**–**k**) The output piezoelectric voltage of three subjects when elbow bending angle changes.

The posture of the arm during spiking is one of the key technical actions that determine the quality of the spike. Figure 4 shows the practical application of the sensor in monitoring the bending angles of knuckles and elbow joints of a volleyball athlete [37]. Figure 4a illustrates the technical essentials of the arm in a spike. Upon hitting the ball, athletes need to straighten their arm and hit the ball immediately to ensure that the ball can be hit from the highest point. At the same time, the palm of the athlete is in the shape of a spoon to fully wrap the ball. In Figure 4b,c, the flexible sensors are attached to the knuckles and elbow joints for measurement. Figure 4d(ii) shows the correct bending angle of the palm while spiking and two wrong palm bending angles for a spike (too large in Figure 4d(iii) and too small in Figure 4d(i)). These two situations can cause the ball to be launched at the wrong angle or slowly (failure of attack).

Three volunteers have participated in this experiment (subjects 1 and 3 are males, and subject 2 is female). Taking subject 1 as an example, in these three cases (one correct and two wrong palms), the output piezoelectric voltage value is 0.044 V, 0.106 V and 0.141 V, respectively (Figure 4e). Similar to the test result in Figure 2, the output voltage increases with increasing bending angle. Therefore, by simply observing the piezoelectric voltage value output of the sensor, the correct spiking action of the test subject can be evaluated. This provides great convenience for the monitoring of athletes in volleyball matches or daily training. Similar results are shown in Figure 4f,g, further illustrating the effectiveness and accuracy of the sensor.

Figure 4h presents the schematic diagram of the arm while straight or not spiking. When the arm is straight, the athlete can hit the ball at the highest point (Figure 4h(iii)), which is conducive to breaking through the opponent’s block defense and finishing an excellent offense [37]. On the contrary, if the arm is not straight, it hinders the athlete’s normal exertion (Figure 4h(i,ii)), causing failure and even injury [38]. As is shown in Figure 4i, in these three different situations, the output piezoelectric voltage value is 0.215 V, 0.343 V and 0.497 V, respectively. The piezoelectric voltage has a positive correlation with the bending angle of the elbow joint. The consequence in Figure 4j,k also shows the same tendency.

**Figure 5:**
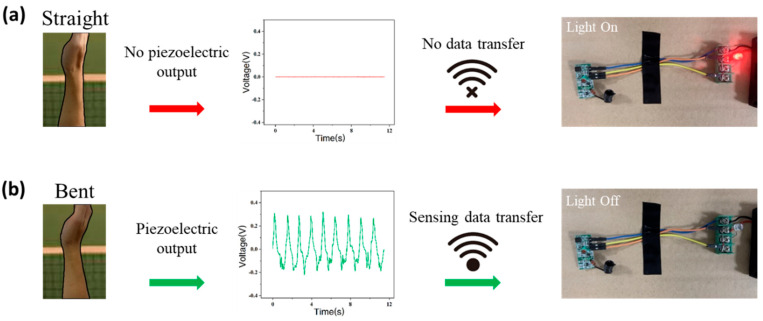
Simple wireless system integrated with the sensor. (**a**) The wireless system when wrist is straight; (**b**) The wireless system when wrist is bent.

Figure 5 demonstrates that the piezoelectric signal generated by the sensor can be wirelessly transmitted to external equipment through a simple wireless transmitting and receiving device. Initially, as the wrist is straight, the LED light on the wireless receiving end maintains a constant light state due the absence of piezoelectric output (Figure 5a). The piezoelectric voltage output generated by bending movement of wrist within 60 s continuously charges a capacitor and connects to the transmitting end, and the LED light on the receiving end is turned off (Figure 5b). The experiment proves that the sensors can wirelessly transmit sports data to external platforms to build big sports data. The sensor focuses on the front-end acquisition and collection of the big data platform. The sports data collected by the sensor can be processed to provide guidance for volleyball player’s daily training, which is also the direction of the sensor’s future development. Compared with the traditional method of using high-speed cameras for video recording, the cost of self-powered sensors is much lower, and will not be affected by many environmental factors. Additionally, the feature of facilitating the collection of sports information makes the sensor very helpful in the construction of big sports data. It may be a new option that the coach and athlete can take into consideration.

## 4. Conclusions

In this paper, a self-powered wearable sensor for monitoring spiking gestures has been presented based on the piezoelectric effect of PVDF film. The sensor has great flexibility and can be fully attached to the surface of the human body. The sensor is powered by collecting body mechanical energy, and the outputting voltage can be treated as a sensing signal of the bending angles of knuckles and elbow joints. The energy generated by the sensor can charge the capacitor and then power wireless electronic devices for building big sports data. Moreover, the sensor can also monitor the pulse changes and language during a volleyball match in real-time. This self-powered wearable gesture-monitoring technique can provide new opportunities for exercise monitoring and promote the development of big sports data.

## Data Availability

The experimental data is contained within the article.
